# The Nature and Treatment of Gout

**Published:** 1860-11

**Authors:** 


II.—*The Nature and Treatment of Gout and Rheumatic Gout.* By Alfred Baring Garrod, M.D., F.R.S., Fellow of the Royal College of Physicians; Physician to University College Hospital; Professor of Materia Medica, Therapeutics, and Clinical Medicine at University College. London: Walton & Maberly, 1859. 8vo., pp. 601.

Two subjects of reflection are suggested by this book before opening its pages. One of these has reference to the multiplication of works treating of individual diseases, or of particular classes of disease. This is a natural effect of the accumulation of practical knowledge. Distinct volumes are now needed for the satisfactory exposition of subjects which formerly claimed only a few pages. This necessity for monographs must become more and more imperative as our knowledge of practical medicine increases. The systematic treatises on the practice of medicine, embracing within the compass of one or two good-sized volumes all that it was important for the practitioner to know of the morbid anatomy, pathology, diagnosis, and treatment of all diseases, have had their day; we doubt if any future bibliographer will have the hardihood to undertake such a work. The other reflection relates to the progress made within the last few years in our knowledge of the relations subsisting between certain diseases and diathetic conditions. This reflection is naturally suggested by a work on gout, from the pen of Dr. Garrod, whose name, for several years past, has been identified with this disease by means of researches which have appeared to establish a pathological connection between the manifestations of the disease and the presence of an excess of the urate of soda in the blood. Assuming this connection to be established, not only have we gained an important increase of our knowledge of gout, but we have encouragement to hope that future researches will lead to the discovery of analogous relations to blood-changes in various affections, as they have with respect to affections dependent on the accumulation of urea in Bright's disease, and, perhaps we may add, the dependence of acute rheumatism on the presence of lactic acid in the blood. As our knowledge advances in this direction, we may expect to understand the origin of many diseases which, in our ignorance, we have been obliged to consider as spontaneous.

With these preliminary reflections we have read Dr. Garrod's work on gout; and we propose to present to the readers of the MEDICO-CHIRURGICAL REVIEW an analysis of those portions of the work which embody the original researches and the experience of the author with respect to matters of practical importance. A limited space being assigned to us, we shall forego much discussion of pathological questions and confine ourselves mainly to the purpose just noted.

The first three chapters, constituting one-sixth of the volume, are devoted to the past literature of the subject, the nature and situation of gouty deposits, and the symptomatology of the disease. We pass by these, simply giving the classification of gout which the author adopts. With a view to simplification, he considers that the phenomena of gout for all practical purposes may be grouped under the heads of—

"1st. Regular gout, (acute or chronic,) consisting, for the most part, of a peculiar inflammation of the structures within and around one or more joints 2d. Irregular gout, which may be manifested either in severe functional disturbance of any organ, or in the development of inflammation in parts other than those connected with the joints. This last variety would therefore include the various forms classed by different writers under the head of atonic, misplaced, retrocedent, lurking, ab-articular gout, etc."

The condition of the blood in gout is the subject of the fourth chapter. The presence of uric acid in excess in this fluid is the first of a series of data for determining the pathological character of the disease. To Dr. Garrod is due the credit of first demonstrating this organic principle in blood taken from a gouty patient. This was in 1847. In 1848 he reported the results of his observations to the Medico-Chirurgical Society, and at that time he appears to have thought that the presence of this principle in the blood, in any quantity, was abnormal. He has since, however, ascertained that, by manipulating with great care, traces of uric acid are determinable in health. Its presence in abnormal quantity is then a proper expression of a pathological condition which he has not found wanting in at least a hundred cases of gout, in which he has examined the blood. Here, then, we have an important fact which is to be associated with other facts in order to determine its pathological import.

The author indicates a mode in which an excess of uric acid in the blood may be readily ascertained with sufficient exactness for ordinary clinical purposes. This is by a process which he terms the "uric acid thread experiment." From one to two fluid drachms of the serum of the blood is to be put into a glass dish, or capsule, which should be broad and flat; to this ordinary strong acetic acid is to be added in the proportion of six minims to each fluid drachm of serum. When the fluids are well mixed, a very fine thread is to be introduced. The glass is then to be put aside, in a moderately warm place, until the serum is quite set and almost dry. If uric acid be present in morbid excess, it will crystallize, and the crystals will be attached to the thread as sugar-candy is formed on a string. Some precautions are to be taken, which the author points out. If the experiment succeed, he may know, not only that uric acid is present in the blood, but present in an abnormal quantity. The experiment is sufficiently simple, and may be resorted to when it is desirable to determine an excess of uric acid in the blood with reference to diagnosis. But if blood-letting be not indicated as a remedy, there is the inconvenience of being under the necessity of practicing a small venesection for the purpose of the experiment. To obviate this inconvenience, the blood may be procured by drawing a small blister, Dr. Garrod's observations showing that an excess of uric acid is contained in the serum thus obtained, provided the blister be not applied to an inflamed surface.

We have said that an excess of uric acid in the blood is the first of a series of data for determining the pathological character of the disease. As we shall proceed to show, this fact is considered in its relations to other facts, so as to reach by a mode of reasoning, at once simple and logical, certain important conclusions. To have made, however, this first step in the investigation more secure, we could have wished that the author had given the results of examinations of the blood for uric acid in cases of different diseases. He gives subsequently the results of examinations in a large number of cases of acute rheumatism, the results being uniformly negative—a very important point, as bearing on the question of identity or non-identity of gout or rheumatism. But is an excess of uric acid in the blood peculiar to gout? May it not also characterize other affections? These questions have an obvious significance, and it would have strengthened the chain of reasoning by which the author reaches his conclusions, had he answered them by giving results of blood analyses in a sufficient number of diseases.

The urine in gout is considered in the fifth chapter. The opinions of writers show considerable discrepancy as to the occurrence of the urates in the urine of gouty patients. The importance of determining whether they are abnormally increased or diminished, taken in connection with the excess of uric acid in the blood, is at once apparent. This point constitutes the second step in the logical sequence of facts relating to the pathology of gout. Dr. Garrod presents, first, a series of examinations of the urine in patients suffering from acute gout, in whom the general health had usually been good during the intervals of the paroxysms. The results show the daily excretion of uric acid, during a fit of gout, to be not necessarily increased, but often notably diminished. Next, he shows by the results in another series of examinations, that the uric acid is diminished in subjects affected with chronic gout, the majority, at the time the examinations were made, having no very urgent symptoms, but suffering from the sequelæ of the disease. In these cases a small amount of albumen was frequently found. Finally, the urine of individuals who had suffered more or less frequently from attacks of gout of varying degrees of intensity, examined at the time of complete freedom from the disease, presented in no case an amount of uric acid exceeding the healthy average, and in the majority it was far below this average. From these facts the conclusion is drawn, that the kidneys lose, to a greater or less extent, the power of excreting uric acid during an attack of acute gout, in the chronic form of the disease, and in the intervals of the paroxysms. In these facts we have an explanation, in part at least, of the accumulation of uric acid in the blood. It accumulates because it is insufficiently eliminated by the organs which are depurative as respects this excrementitious principle. It is a curious fact, that the impaired excretory power of the kidneys in this disease relates to uric acid exclusive of urea; the urea-eliminating faculty remains comparatively intact.

The author next enters upon the morbid anatomy of gout. The sixth and seventh chapters are devoted to this subject. Premising here, as in entering upon the consideration of other subjects, a historical sketch of the observations and opinions of preceding authors, he proceeds to lay before the reader the results of numerous autopsies made by himself. He considers, first, the alterations seen in and around the articulations. These were observed in the following classes of cases: *First.* In subjects of chronic gout, with extensive chalk-stones. *Second.* In subjects with no appreciable deformity and no visible deposits of chalk-stones, except one or more specks on the cartilage of the ear. *Third.* In subjects i[illegible]n no trace of chalky matter was externally visible in any part. *Fourth.* In one subject in which only the ball of the right great toe had been affected with gouty inflammation. In the *first* class of cases there was more or less complete incrustation of the surfaces of the bones with white matter, together with infiltration of the surrounding ligaments, deposit within the tendons and their sheaths, and occasionally along the tendinous expansions of the muscles. He has never found the deposit within the substance of bones, although its occurrence in this situation is asserted by some of the French observers. In the *second* class of cases, many of the joints were affected, showing that extensive deposit may take place internally without corresponding external development. In the *third* class of cases, joints were found to be incrusted, although there was no evidence of this afforded by examination during life. In the single case embraced under the *fourth* class, the articular cartilages of the metatarso-phalangial joint were incrusted with the white deposit, and the surrounding ligamentous tissues freely sprinkled with it. The deposit in all these cases was shown to be urate of soda by the formation of the muroxide or the purpurate of ammonia, and by the crystalline forms under the microscope. The latter appearances are illustrated by beautiful drawings. A similar deposit never occurs in acute or chronic rheumatism, nor in the affection known by the name of chronic rheumatic arthritis.

What relation has the deposit of the urate of soda within and around the articulations to the gouty inflammation of the parts? Dr. Garrod believes that the former stands to the latter in the relation of cause rather than effect; in other words, the deposit of the salt first takes place, and by its presence gives rise to inflammatory action. This conclusion, which he subsequently discusses at greater length, holds an important place in the series of data on which the pathology of the disease is based.

Dr. Garrod shows that the deposit of the urate of soda does not, as has been imagined, replace the cartilages supposed to have become absorbed. The deposit is interstitial, extending only a small distance; it may be removed by digesting the part in rain water for some days, and the end of the bone then presents an almost healthy appearance.

The morbid changes in the kidneys of gouty subjects have an important bearing on the pathology of the disease. Dr. Garrod's observations show that the renal organs become seriously implicated in even the slighter forms and early stages of gout. In advanced disease they are considerably contracted, the decrease of size being chiefly at the expense of the cortical portion, which is sometimes so wasted that the pyramids almost reach the surface of the organ. This condition is by no means distinctive of gout; it is found in connection with other diseases. An appearance, however, which, so far as Dr. Garrod's observations have extended, is distinctive, is due to a deposit of a chalk-like substance in the form of streaks or white lines, chiefly in the direction of the tubes of the pyramidal portion. The mammilla of each cone, also, in some cases presented an appearance of white points, from the deposit of the same matter in this situation.

The deposit causing these white streaks and points is the urate of soda, which here, as elsewhere, presents under the microscope its crystalline forms. Dr. Garrod thinks that the deposit is not within the tubuli uriniferi, but that it is imbedded in the fibrous structure. The presence of this deposit in the kidneys, in greater or less quantities, characterizes all the cases of gout in which autopsies were made by Dr. Garrod, with the exception of a single case, in which the patient had only had two attacks of the disease, the affection in each attack being confined to the ball of one great toe. The contraction of the kidneys in persons who have suffered much from gout, Dr. Garrod attributes, with Dr. George Johnson, to disintegration and removal of the epithelium of the tubes. As a result of this, the tubes gradually become wasted and shriveled from the collapse of the surrounding tissue. This condition of the kidney occurs in advanced gout, and constitutes the so-called gouty kidney of Dr. Todd and Dr. Johnson.

The causes of gout are not only of great importance in their direct practical bearing on the management, but they are to be considered among the data on which a correct understanding of the pathology of the disease must rest. Chapter eight is devoted to this subject. Among the predisposing causes, hereditary influence is considered as holding the first rank. The disease is comparatively rare among females. Age exerts a decided influence. Dr. Garrod has never seen a true case much under twenty. In by far the majority of cases, the disease makes its appearance in adult age after the growth of the body is complete and before the powers begin to decline, or between the thirtieth and fortieth year. Dr. Garrod does not attach much importance to a particular temperament, concluding, as we think very correctly, that this term is often employed in medicine with very little precision. Next to hereditary influence, the most important predisposing cause is the influence of fermented and distilled liquors. But all kinds of liquors are not equally operative in predisposing to the disease. Distilled spirits, when exclusively taken, appear to exert little or no power in inducing gout; whereas the reverse holds good with wines, strong ales, and porter. This may explain the comparative infrequency of gout in Scotland, Ireland, and perhaps, also, in this country.

With reference to the influence of malt liquors, the author quotes the following interesting account by Dr. William Budd:—

"There is a body of men employed in the Thames, whose occupation it is to raise ballast from the bottom of the river; as this can be done only when the tide is ebbing, their hours of labor are regulated by this circumstance, and vary through every period of night and day. They work under great exposure to the inclemencies of weather; their occupation requires great bodily exertion, occasioning profuse sweating and much exhaustion. In consideration of this, their allowance of liquor is very large; each man drinks from two to three gallons of porter daily, and generally a considerable quantity of spirits besides. This immoderate consumption of liquors forms the only exception, as far as relates to food, which these men offer to the general habits of the lower classes, and, although not a numerous body, many of them are yearly admitted to the seamen's hospital ship, affected with gout. This is a very interesting fact, and seems to show that no amount of bodily exertion is adequate to counteract the influences of such large doses of porter; the exposure of ballasters to wet seems to favor its operation. These men are almost all derived from the peasantry of Ireland; they can rarely, therefore, inherit a disposition to gout."

In a large proportion of cases of gout, dyspeptic ailments are connected with the disease. These may be concerned in the production of the uric acid diathesis, or they may be effects of this diathesis. The more prominent of the symptoms of the dyspepsia connected with the diathesis are thus summed up:—

"Heart-burn and eructations, oppression, and frequently sleepiness after food; a feeling of distention in the epigastrium, sometimes accompanied with tenderness; some fullness over the hepatic region, and the edge of the liver often projecting a little below the ribs, and occasionally tender to the touch; the tongue much furred, red at the tips and edges; a disagreeable and clammy taste in the mouth, and the saliva and buccal secretion occasionally more adhesive than natural. The bowels are usually more or less confined, scybalous, sometimes very dark in appearance, and at other times light and clay colored, showing a defective secretion of biliary matter. Accompanying these symptoms there is generally a scanty secretion of urine, which is high colored and acid, and often giving rise, on cooling, to a copious deposit of urates, or to a sediment of crystalline uric acid, varying from a brick-dust red to a pale yellow."

This group of symptoms occurrs so often without the development of gout, that we are hardly warranted in placing much confidence in them as premonitions, unless the disease have been already experienced; and on the other hand, an attack of gout not unfrequently occurs without any marked premonitions.

Severe study and mental anxiety, venereal excesses, and other powerfully depressing influences appear to be often instrumental in engendering a gouty habit. The disease prevails less in hot than in temperate climates. Attacks are most liable to occur in the spring months.

Dr. Garrod has been led to regard the influence of lead as a predisposing cause, by finding that of the patients coming under his care with the disease in University College Hospital, at least one in four had, at some period of their lives, been affected with lead poisoning. Another fact leading to this conclusion is an excess of uric acid in the blood in cases of lead poisoning, not only in those who have previously suffered from gout, but even where no symptoms of the latter disease had ever shown themselves. Uric acid, however, is only present in excess in certain cases of lead poisoning.

Among the exciting causes of gout, the author mentions, as the most important, indulgence in alcoholic beverages, indigestion, cold and moisture, severe mental or bodily labor, local injury of joints, and the occurrence of hemorrhage. He has seen a fit of gout produced by copious hematemesis, and by the loss of blood from a tooth and from the nostrils, as well as other forms of hemorrhage.

Having considered the subject of causation, the author, in the succeeding chapter, discusses the pathology or nature of gout. That the pathology of the disease involves an excess of uric acid in the blood has been already foreshadowed. It has been seen that the urine contains less of this principle than the healthy average. The author's researches in the morbid anatomy of the disease have also shown that a deposit of the urate of soda takes place always within or around the joints, and that probably this deposit precedes the development of gouty inflammation. Now, then, the questions arise, is the abnormal quantity of uric acid in the blood a result of the excessive production of this principle which exists in minute quantity in health; or does the uric acid accumulate in the blood, without any excess of production, simply because it is not adequately excreted by the kidneys; or may its excess in the blood be owing to an excessive production and an insufficient excretion combined? Dr. Garrod continues to hold to the views enunciated by him in a paper published in the *Medico-Chirurgical Transactions* for 1848. According to these views, the excess of uric acid in the blood may be due either to the uric acid excreting function of the kidney being defective, or to an undue function of the urate of soda in the blood; the latter, in fact, involving the former. The presence of uric acid in an abnormal quantity in the blood, however, does not fully explain the occurrence of a gouty paroxysm. The latter may be induced by a greatly-augmented formation of the acid, or by the eliminating power of the kidneys, being unusually checked, or *by a less alkaline condition of the blood.* We have italicized the latter part of the preceding sentence, because it relates to a point not before referred to. Diminution in the alkaline state of the blood lessens its power of holding urate of soda in solution, and hence promotes its deposition, especially in tissues in which the reaction is naturally less alkaline than in blood. We quote the author's remarks on this point:—

"To illustrate this experimentally, we may take a watery solution of the common phosphate of soda and dissolve uric acid in it, but only to such an extent as to insure the fluid remaining alkaline. Any diminution of this reaction by the subsequent addition of a weak acid will cause the precipitation of the urate of soda; but if the fluid be rendered acid, crystallization of free uric acid will ensue."

With regard to the phenomena of a fit of gout the author continues:—

"It is essential for its production that there should be an impure condition of the blood from the presence of urate of soda, and such impurity may be produced by the action of any of the various predisposing causes of the disease, and is probably of itself sufficient to give rise to the premonitory symptoms which are chiefly manifested in functional derangement of the digestive and circulating organs. To excite a gouty paroxysm, however, requires the additional operation of other agents, namely, those causes by which the urate of soda is either suddenly augmented or rendered less soluble, and its crystallization consequently induced in the ligamentous and cartilaginous structures of the joints."

The conclusions presented in the previous chapters of the work have led, as it were step by step, to the general views of the pathology of gout expressed in the foregoing quotations. In leaving this subject we have only to say that the pathological views of the author seem to be logically proven, and if not correct, they are to be disproved by researches which will show error in some of the premises. Assuming these to be free from error, the reasoning is complete.

The successful management of gout depends in no small measure on correct views of its pathology. This is only applying to this particular disease a remark more or less applicable to all diseases. The remark does not undervalue clinical experience. The latter must be guided to a considerable extent by pathological views; and practical medicine will become as perfect as possible when all that can be educed from clinical experience is conjoined with all that can be ascertained respecting the pathology of diseases. The period when these requisites for perfection are complied with will not arise in our day. In entering on the subject of the treatment, Dr. Garrod takes exception to Cullen's aphorism of trusting to patience and flannel. He believes that a fit of gout is as much under the influence of remedies as any other inflammatory affection, and that the more chronic forms are greatly within the power of the physician. During an acute attack the diet should be strictly antiphlogistic, and so long as febrile movement is present the patient should keep the bed. The constitutional treatment is to be directed toward the diminution of inflammation and febrile disturbance, and the restoration of the blood to a healthy condition. Purgatives he considers as possessing no peculiar power of lessening the articular inflammations; the secretions from the mucous membrane of the bowels, even when hydragogue cathartics are given, do not contain a sufficient amount of uric acid to relieve the overloaded state of the blood. Purgative remedies are only called for when indicated by the undue retention of excrementitial matters in the intestines. Mercury does not exert any specific control over gouty inflammation, and hence should only be administered as a purgative. In advanced gout the use of mercury is to be avoided. Diuretic and diaphoretic remedies are often of much service. The saline diuretics are serviceable also in rendering the blood more alkaline. The acetate of ammonia is mentioned as a suitable remedy to induce diaphoresis, its action being aided by the free use of diluents. The author's experience has led him to withhold opium unless the pain be excessive. Bleeding is not curative, and the only advantage which can possibly be derived from its use is the relief of plethora. Whenever carried to an extent to cause subsequent depression, the effect is to render the attack more frequent and prolonged. The only local application required in the majority of cases is cotton wool covered lightly with oiled silk. Anodyne embrocations are occasionally useful. The author does not mention hypodermic injections which suggest themselves as likely to prove useful by way of palliation of pain, to say the least. We have found methodic friction of the affected joints, even during the height of the inflammation, extremely effective. Commencing with the gentlest friction, the force used may be gradually increased until at length strong pressure is borne without suffering, and the affected joint is greatly relieved.

The use of colchicum is deemed of sufficient importance to devote to its consideration an entire chapter. That this remedy has a most powerful influence upon the progress of gouty inflammation he deems undeniable, and he considers that its action is not limited singly to the removal of gout when it attacks the joints, but that it proves efficacious in its masked and irregular forms. The author coincides with Sir Henry Holland in saying that we may sometimes diagnose gout from other forms of inflammation by noting the influence of this drug. The controlling influence of the remedy is independent of its purging properties, and it is not necessary to carry it to the extent of purging. Nor does its value depend on its effecting an elimination of uric acid through the kidneys, for in a series of cases in which the urine was examined while patients were using the remedy, the amount of uric acid in that fluid was not increased. Moreover, it acts beneficially often when it does not act as a diuretic, the quantity of urine being in some instances lessened. In short, Dr. Garrod does not attempt to explain the *modus operandi*, but rests his opinion of its efficacy exclusively on clinical observation, which he thinks affords evidence that it possesses as specific a control over true gouty inflammation as cinchona barks over intermittent diseases. When prescribed with due care, he believes that it has no tendency to prolong the gouty paroxysm, or render the disease more chronic in its character.

In the chronic stages of gout the inflammatory state of the joints may be relieved by colchicum administered in very minute doses, and continued only for a short period at a time. The iodide of potassium and guaiacum may also be resorted to with advantage. Dr. Garrod does not find the iodide of potassium to possess any appreciable power to remove the deposits of the urate of soda, and he thinks that all the good results of this remedy may be obtained by giving from half a grain to a grain twice or three times daily. We must express surprise at the statement just made; we can hardly conceive of such small doses exerting any effect save as a placebo.

But in chronic gout the grand indication is to remove the abnormal state of the blood. Bearing in mind that the excess of urate of soda in the blood depends on undue formation on the one hand, and on the other hand, on deficient excretion from the renal organs, the indication just stated is to be fulfilled by different measures of treatment. To prevent the undue formation of uric acid, the diet and regimen are to be regulated, and the deficient excretion is to be controlled by remedial agents. The remedies best adapted to purify the blood are those which increase the activity of the secreting organs, and also those which prevent the deposit of the urate of soda in the tissues, as well, perhaps, as remove it when already infiltrated. These objects call for alkalies and salines. Among the alkalies are to be included such neutral salts as the citrates and tartrates which are decomposed in the blood and render the urine less acid in reaction. In administering alkalies, it is to be borne in mind that they are to be introduced into the system. Hence the liquor potassæ or the liquor sodæ are not appropriate, the influence of these being chiefly limited to the stomach and its contents. The carbonates and bicarbonates of these alkalies are preferable, having the effect to render the blood more alkaline, and to alter the secretion of the kidneys, these effects being produced especially by the bicarbonates. The phosphate of soda has the same effects. With reference to rendering the uric acid of the blood and urine more soluble, the potash salts are to be preferred to the corresponding salts of soda; they also act more powerfully upon the kidneys as diuretics. The fact of the geater power of the potash salts in imparting solubility to uric acid may be illustrated experimentally by placing in solutions of the carbonates of potash and soda respectively, small portions of gouty cartilage infiltrated with the urate of soda. The author states that after the portion treated by the potash solution has become quite free from the urate, the portion immersed in the soda remains almost in its original state.

The author is in the habit of prescribing alkaline remedies in the treatment of the paroxysms of gout, giving preference to the bicarbonate or the citrate, or the acetate of potash, in consequence of the greater solvent power of these salts for uric acid as compared with the soda salts, and their greater diuretic effect. But he attaches far more importance to the continuance of these remedies when there is no inflammatory joint affection present; they should be given in small doses, repeated two or three times a day, in a very dilute form, always on an empty stomach, a little while before taking food. Much importance belongs to these points. With reference to the success of these remedies the author says:—

"I have repeatedly given them to patients who had for many years suffered annually from numerous severe attacks of the malady, with the effect of keeping them free from its return for a whole twelvemonth, and this exemption was accompanied with a decided improvement in the general health. Upon the whole, I have found no treatment so efficacious, and it is attended with this great advantage, that it can scarcely be regarded in the light of drugging, as no really foreign body is thereby introduced into the economy, but merely a slight increase of those saline matters which are present in many articles of food, normal to the constitution of the blood and even necessary to its healthy constitution."

The author has of late made trial with the carbonate of lithia in several cases of chronic gout with advantage. By experimenting with a solution of this salt in the same way as with solutions of the carbonate of potash and soda, in placing in the solution a piece of cartilage infiltrated with the urate of soda, he found that the deposit was more quickly removed than when the potash solution was used. The carbonate of lithia should be given freely diluted in common water or aerated water.

The phosphate of ammonia, first introduced as a remedy for gout by Dr. Buckler, of Baltimore, is mentioned, but the author has evidently not given it much trial. This remedy was suggested as applicable on chemical grounds, the ammonia being supposed to combine with the uric acid, and the phosphorus with the soda, forming two soluble compounds which would be readily eliminated by the kidneys. As Dr. Garrod states, this remedy has been employed by several physicians with success.

Dr. Garrod's experience with the sulphate of quinia is, that it is in no sense a specific, as some have contended, yet he has often seen remissions of the paroxysm follow its use. The ferruginous tonics when indiscriminately given are likely to excite a paroxysm, and for the most part are contra-indicated. When iron is called for he prefers either that reduced by hydrogen, or the saccharine carbonate, or the citrate. It is hardly necessary to add, that regulation of the diet and regimen is of essential importance in the management of chronic gout; but we are glad to see that in his excellent remarks on this subject Dr. Garrod points out the error of carrying too far a reducing plan of dietetics. We could cite an instance from our own experience in which we believe the life of a valued friend was sacrificed to a determination to eradicate in this way the disease.

In concluding the consideration of the treatment, Dr. Garrod devotes a chapter to the subject of mineral waters. His remarks on this subject strike us as eminently judicious, but it is difficult to present an abstract of them within the limits to which we are confined. He attributes their efficacy in a measure to one action common to all kinds, viz., the influence of the water itself. We believe him to be correct in this remark, and it is a point not sufficiently appreciated. Water, as he justly says, when absorbed in large quantities, stimulates powerfully the processes of the animal economy and increases the various secretions. Much of the benefit derived from mineral springs, he doubts not, might be attained by ingesting the same amount of pure water under similar circumstances. With respect to the Vichy waters, after having witnessed the plan of treatment adopted at the baths, he arrives at the conclusion that they are undoubtedly potent agents, but capable of causing much evil as well as effecting good. He regards them as injurious in chronic cases when the system is already lowered. Containing, as they do, the carbonate of soda, they tend to diminish rather than augment the solubility of the urate of soda. They are useful in strong and robust subjects during the complete intervals of acute gout. We pass by remarks on other waters which, in this part of the world at least, are less known.

We come now to the last two chapters of the work. The first of these is devoted to the consideration of the irregular forms of gout. We are glad to find the opinion expressed by so high an authority as Dr. Garrod, that the numerous forms of gout described by many writers, are nothing more than coexisting diseases. To call pleurisy, bronchitis, etc., in certain cases, gouty affections, is certainly a misnomer, unless it be proved that these local affections are due to the same internal cause which determines the peculiar inflammation within and about the joints which characterizes gout. The habit of considering all affections as gouty when they occur in connection with symptoms appearing to point to the gouty diathesis, or when they are developed in persons subject to attacks of gout, not only involves a pathological error, but may lead to erroneous practice. The term irregular gout, moreover, as the author intimates, is often used as a cloak for ignorance, when the true nature and source of ailments are not understood. Still, it is not to be doubted that the diathesis which determines regular gout may give rise to symptoms aside from those relating to the joints. How are we to decide when these are of a gouty character? With reference to this question in individual cases, the existence of hereditary influence, the age and the sex of the patient, are to be considered. The previous existence of any joint affection is to be inquired into, and its character ascertained. In important cases of doubt the blood is to be examined for an excess of uric acid, obtaining it by a small venesection, or by testing the serum from a blister in the manner already described.

Dr. Garrod does not deny the occurrence of retrocedent gout in the stomach, but he states that no case has ever fallen under his observation. He doubts, however, the occurrence of gastric inflammation under these circumstances; the symptoms and the relief afforded by stimulants show the affection to be functional. Cases have been reported in which, coincidently with the disappearance of the joint affection, the heart became greatly distended; but there is no evidence that there ever occurs a metastasis of the inflammation to this organ. When apoplexy supervenes on the cessation of the gouty affection, it is probable that the brain was previously diseased. In all these cases, in addition to such remedies as may be indicated by the symptoms referable to the suffering organs, it is proper to apply warmth and counter-irritation to the extremities, with a view of bringing back the inflammation to these parts.

Gouty affections referable to the digestive organs are often dependent on the same cause which leads to the fit itself, viz., an impure state of the blood from the presence of the urate of soda. Cough and asthmatic breathing appear to be frequently produced by a gouty state of the system. Dr. Garrod's observations, showing the almost constant deposit of urate of soda within the kidneys, (to which reference has been made,) lead him to think that true gouty inflammation is often set up in the structure of these organs. Gouty ophthalmia and iritis admit of doubt; but in this connection may be mentioned the frequent occurrence of little nodules of urate of soda upon the cartilage of the ear. These are noticed in former parts of the work, but have not before been distinctly referred to in this review. Among phenomena referable to the venous and muscular systems, Dr. Graves noticed in certain gouty subjects an insufferable desire to grind the teeth, appearing to originate in a disagreeable, uneasy sensation in the teeth themselves. We have noticed this in the case of a patient affected with gout. Neuralgia is not an uncommon manifestation of gout, occurring in various situations. Hysteria and mania seem to be sometimes attributable to the gouty state. In these and other affections, when they are really irregular forms of gout, the colchicum will be likely to prove a valuable remedy.

The last chapter of the work is occupied mainly with rheumatic gout, and an affection which the author styles rheumatoid arthritis. The term rheumatic gout is often very loosely applied; and when we consider the points of difference between gout and rheumatism, the question which the author asks may well enough be put, is there a disease to which the term *rheumatic gout* can properly be applied? In answer to this question, he expresses the conviction that no combination of the two diseases is ever seen in nature. He is of opinion that this term is applied incorrectly sometimes to cases of true gout when the affection has traveled above the knees and invaded the upper extremities; in other cases to chronic or subacute rheumatism, but particularly to a disease having a peculiar pathology, in no way related to gout, and not necessarily to rheumatism, to which he gives the name of rheumatoid arthritis. In the diagnosis of gout, in addition to points usually given, and with which our readers are presumed to be familiar, Dr. Garrod impresses the importance of an examination of the blood or serum; an excess of uric acid being constantly present in gout, and never in rheumatism. He also suggests an examination of the external ear, the presence of a spot or two on the helix, due to the deposit of the urate of soda, leading quickly to a correct diagnosis in some cases.

Rheumatoid arthritis, called by Dr. Adams, of Dublin, chronic rheumatic arthritis, and by Dr. McLeod capsular rheumatism, included by Fuller and others under the head of rheumatic gout, is an affection characterized by pain in certain joints, the wrist, fingers, or hips; attended at first by little or no swelling, but after a time by enlargement, from secretion of fluid in the articular cavities, as determined by softness and fluctuation to the touch. After absorption has taken place, the swollen joints become gradually hard. There is but slight pain and little constitutional disturbance, unless a large number of joints are implicated. A chronic form of the disease, principally affecting the smaller joints, and especially the last phalanges of the hand, is frequently met with. The morbid anatomy differs essentially from that of gout, in the absence of deposits of the urate of soda. Instead of this, ulcerations of the cartilages and changes in the bony structure ensue. These effects neither occur in gout nor in rheumatism. The blood in patients affected by this disease does not present an excess of uric acid, nor is there any specific alteration in the urine.

Finally, with respect to the prognosis in gout, it is always favorable, so far as concerns immediate danger from a paroxysm. Dr. Garrod remarks that he has never known or heard of an instance which terminated fatally. But he concurs with others in repudiating a common belief that the occurrence of gout is desirable because it cures other ailments, and frees the system from any lurking malady. The endeavor to induce an attack of gout is to be deprecated. The disease, in fact, has a tendency to shorten life. When once experienced, it will be likely to return after longer or shorter intervals. There are very few exceptions to this rule. Gouty patients, however, may live to a good old age. The prognosis is less favorable in proportion as deposits have occurred, crippling the joints, and the kidneys have lost their uric acid excreting power, the urine at the same time containing albumen. The examination of the urine is thus of great value as an aid to prognosis. We give the two concluding paragraphs of the work :—

"In concluding my remarks upon this subject, I may state, that I consider even a single fit of gout, however slight, should be looked upon as an intimation that the patient cannot go on with impunity in his then habits of living; a warning that either he must change them, or expect returns of the disease, which are certain to increase both in frequency and duration, and likely, if no heed be taken, to embitter and shorten existence."

"On the other hand, I am equally persuaded that if proper regimenal and medicinal precautions be taken, the gouty patient may be saved from such a termination, and that the disease, in place of increasing in severity, may be gradually mitigated, and probably interfere but little with the comforts of life."

We have aimed in this analytical review of Dr. Garrod's work to present, as compactly as possible, the substance of the author's researches and conclusions. We have not found the task wearisome. We have rarely read a work which has afforded us greater satisfaction, and we have derived renewed pleasure in making an abstract of its contents. We trust that we have succeeded in our undertaking sufficiently, at least, to lead our readers to desire to examine the work for themselves. In the arrangement and handling of his subjects, the simplicity and clearness of style, and the philosophic spirit which pervades its pages, we regard it as a model book.

